# Ivermectin suppresses tumour growth and metastasis through degradation of PAK1 in oesophageal squamous cell carcinoma

**DOI:** 10.1111/jcmm.15195

**Published:** 2020-03-31

**Authors:** Liang Chen, Shuning Bi, Qiuren Wei, Zhijun Zhao, Chaojie Wang, Songqiang Xie

**Affiliations:** ^1^ School of Pharmacy Henan University Kaifeng China; ^2^ Department of Medicine and Therapeutics Luohe Medical College Luohe China; ^3^ The Key Laboratory of Natural Medicine and Immuno‐Engineering Henan University Kaifeng China

**Keywords:** ivermectin, metastasis, oesophageal squamous cell carcinoma, PAK1, tumour growth

## Abstract

Oesophageal squamous cell carcinoma (ESCC), the most common form of oesophageal malignancies in the Asia‐Pacific region, remains a major clinical challenge. In this study, we found that ivermectin, an effective antiparasitic drug that has been approved for patients to orally treat onchocerciasis for over 30 years, displayed potent antitumour activity against ESCC cells in vitro and in nude mice. We demonstrated that ivermectin significantly inhibited cell viability and colony formation, and induced apoptosis through a mitochondrial‐dependent manner in ESCC cells. Ivermectin also abrogated ESCC cell migration, invasion, as well as the protein levels of MMP‐2 and MMP‐9. Mechanistically, ivermectin strongly inhibited the expression of PAK1; by further gain‐ and loss‐of‐function experiments, we confirmed that PAK1 played a crucial role in ivermectin‐mediated inhibitory effects on ESCC cells. In addition, the data indicated that ivermectin promoted PAK1 degradation through the proteasome‐dependent pathway. Additionally, ivermectin synergized with chemotherapeutic drugs including cisplatin and 5‐fluorouracil to induce apoptosis of ESCC cells. Interestingly, the in vivo experiments also confirmed that ivermectin effectively suppressed tumour growth and lung metastasis of ESCC. Collectively, these results indicate that ivermectin exerts a potent antitumour activity against ESCC and is a promising therapeutic candidate drug for ESCC patients, even those carrying metastasis.

## 1 INTRODUCTION

Oesophageal cancer, the eighth most frequent cancer, is the sixth leading cause of cancer‐related mortalities in the world, with more than 455 thousand people newly diagnosed oesophageal cancer cases and over 400 thousand deaths occurred per year.^1^ The majority of oesophageal cancers can be split into oesophageal adenocarcinoma (EAC) and oesophageal squamous cell carcinoma (ESCC) according to the histological subtypes.^2^ Worldwide, there are an estimated 398 000 new cases of ESCC (representing 87% of all oesophageal cancer) occurred in 2012.^3^ About 80% of the total global ESCC cases occur in the South‐East and Central Asian region, especially in China where contribute 53% of the global cases.^1^ Although the use of diverse strategies including surgical resection, in combination with chemotherapy (cisplatin and 5‐fluorouracil), radiotherapy and other modern techniques, the prognosis for ESCC patients is still poor, with a less 20% five‐year overall survival rate,^4^ mainly due to lacking clinical approaches for early diagnosis, high incidences of tumour metastasis and recurrence, as well as the resistance of the cancer cells to radiotherapy and chemotherapy.^5^, ^6^, ^7^ Thus, there is an urgent need to find or develop novel potential therapeutic agents.

Avermectin (AVM), a 16‐membered macrocyclic lactone compound, was initially discovered and purified from *Streptomyces avermectinius* by Ōmura and Campbell in 1967.^8^, ^9^ Avermectin exhibited remarkably profound antiparasitic bioactivity and earned the 2015 Nobel Prize for Physiology or Medicine. Ivermectin is a dihydro derivate of avermectin that displayed much more efficient against several kinds of parasitic diseases including to onchocerciasis (also known as river blindness) and lymphatic filariasis.^10^, ^11^ Mechanistically, this compound can highly and selectively bind to glutamate‐gated ion channels (Glu‐Cl) or increase the activity of neurotransmitter‐gated gamma‐aminobutyric acid (GABA) only in a broadspectrum of parasites but not mammals.^12^, ^13^ Ivermectin is a well‐tolerated agent that has been approved for application in humans to treat onchocerciasis, strongyloidiasis, parasite infections and other worm infestations including ascariasis, enterobiasis and trichuriasis.^14^, ^15^ In 2010, Sharmeen et al first reported that ivermectin exhibits strong pre‐clinical activity against leukaemia cells and primary patient samples, and diminishes tumour growth in three different mouse models of leukaemia.^15^ Consistently, a recent report showed that ivermectin induces chronic myeloid leukaemia (CML) cell apoptosis, but not normal hematopoietic cells, through inducing oxidative stress and disrupting mitochondrial functions.^16^ Besides haematologic malignancies, mounting evidence has demonstrated that ivermectin is a promising antineoplastic agent for a wide range of malignant solid tumours including breast cancer, epithelial ovarian cancer, melanoma, colon cancer and glioma.^14^, ^17^, ^18^, ^19^, ^20^ Up to date, whether ivermectin is active against ESCC remains underexplored.

In the current study, our goal was to explore the antitumour activity and its molecular mechanism of ivermectin against ESCC. The results showed that ivermectin effectively suppressed ESCC cell growth in vitro and in vivo, and induced apoptosis. Moreover, ivermectin diminished the abilities of migration and invasion, and the metastasis in nude mice. Mechanistically, we found that PAK1 played a crucial role in ivermectin‐mediated inhibitory effects on ESCC cell growth, migration and invasion. Furthermore, ivermectin enhanced the sensitivity of ESCC cells to cisplatin (CDDP) or 5‐fluorouracil (5‐FU). Altogether, our studies provided the first pre‐clinical evidence demonstrating that ivermectin is a promising therapeutic candidate drug for ESCC patients.

## 2 MATERIALS AND METHODS

### 2.1 Reagents and antibodies

Ivermectin (#S1351) was obtained from Selleck Chemicals. MG132 (#ab141003), CDDP (#P4394) and 5‐FU (#V900394) were purchased from Sigma‐Aldrich. Cycloheximide (CHX, #A8244) was from APExBIO Technology LLC. Antibodies against PAK1 (#2602), Raf1 (#9422), MEK1 (#2352), PARP (#9532), phospho‐MEK1 (S298, #9128), phospho‐Raf1 (S338, #9427), Caspase‐3 (#9665), MMP‐9 (#3852), MMP‐2 (#4022), Cleaved Caspase‐3 (#9664), Bax (#5023), Bcl‐xL (#2762), Mcl‐1 (#5453), XIAP (#2042), Survivin (#2808), Cytochrome c (#4272), AIF (#5318) and COX Ⅳ (#4850) were obtained from Cell Signaling Technology. Anti‐Ki67 (#ab15580) antibody was obtained from Abcam. Antibody against Actin (#4700) was obtained from Sigma‐Aldrich. Peroxidase‐conjugated secondary antibodies including Goat antimouse IgG (#ZB‐2305) and Goat anti‐Rabbit IgG (#ZB‐2301) were brought from ZSBG‐Bio.

### 2.2 Cell culture

Human ESCC cell lines (EC109, KYSE70, KYSE150 and KYSE30) and the immortalized human oesophageal epithelial cell line Het‐1A were cultured as previously described.^5^ All cells were tested periodically for mycoplasma contamination and authenticated by using the short tandem repeat (STR) analysis.

### 2.3 Quantitative real‐time PCR (qRT‐PCR)

ESCC cells pre‐treated with increasing concentrations of ivermectin, and then the total mRNAs were isolated by using the TRIzol reagent (Invitrogen) according to the manufacturer's instructions. Reverse transcription was conducted by using 1 mg of total RNA and PrimeScript RT Master Mix (TaKaRa). qRT‐PCR was conducted on an ABI Prism 7, 900 Real‐Time PCR System (Thermo Fisher Scientific) by using SYBR Premix Ex Taq II (TaKaRa). Fold enrichment was calculated performed with the 2^−ΔΔCt^ method. GAPDH served as an internal control. The sequences of primers used for qRT‐PCR were as follows: GAPDH, 5′‐GAAGGTGAAGGTCGGAGTC‐3′ (forward) and 5′‐GAAGATGGTGATGGGATTTC‐3′ (reverse); PAK1, 5′‐CGCAGGCTGTTCTGGATGT‐3′ (forward) and 5′‐GTGGCACTGCAGGAGTCTCA‐3′ (reverse).

### 2.4 MTT assay

MTT assay was used to detect the cell viability of ESCC cells. In brief, two thousand cells per well were plated in 96‐well plates (Corning) and incubated for 24 hours, and then escalating concentrations of the indicated chemicals were added and incubated for 72 hours. Four hours before the end of experiments, twenty μL of MTT reagent (Sigma‐Aldrich) was added. At last, one hundred μL of DMSO was used to dissolve the formazan. Absorbance was recorded by using a Synergy HT Microplate Reader (Bio Tek) at a wavelength of 570 nm. IC_50_ values were assessed by using GraphPad Prism version 7.0 (GraphPad software).

The synergism of ivermectin in combination with CDDP or 5‐FU was assessed by the median‐effect method of Chou and Talalay.^21^ The combination indexes (CIs) were evaluated by using the CalcuSyn software version 2.0 (Biosoft). CI < 1 indicates that the combinational effect between two drugs was synergistic.

### 2.5 Soft agar assay

Soft agar assay was used to determine the anchorage‐independent growth as described previously.^22^ Briefly, upon ivermectin treatment, about 2000 cells per well were placed into 24‐well plates (Corning). Cells were embedded into 0.4% bactoagar supplemented with 10% FBS and layered on top of a solidified 0.8% bactoagar base (Sigma) with 10% FBS. After incubation for 14 days, the colonies containing over fifty ESCC cells were counted with an inverted optical microscope and analysed with GraphPad Prism version 7.0.

### 2.6 Measurement of cell apoptosis

For measuring apoptosis, ESCC cells were pre‐treated with escalating doses of ivermectin for forty‐eight hours or fifteen μmol/L ivermectin for the indicated time periods. Cells were collected and stained by using an annexin V‐FITC Apoptosis Detection Kit (Sigma‐Aldrich) following the manufacturer's instruction. Apoptosis was immediately measured by using the BD FACSVerse flow cytometer and its software.

### 2.7 Evaluation of mitochondrial membrane potential (MMP)

The MMP was measured as previously described.^23^ Briefly, ESCC cells were incubated with 5 μmol/L ivermectin for the indicated times. Cells were then collected and stained with Rh123 (20 μmol/L) at 37°C for 30 min. After washing thrice with 1 × PBS, the MMP of ESCC cells was detected by using the BD FACSVerse flow cytometer and its software.

### 2.8 Immunoblotting analysis

The indicated protein expression was detected by using immunoblotting analysis as previously described.^24^ In brief, the whole cell lysates were prepared in RIPA buffer containing 1 × protease inhibitor cocktail (Roche, Indianapolis, IN). Thirty μg of total proteins was separated by using SDS‐PAGE and transferred onto nitrocellulose membranes. After blocking with 5% non‐fat milk, the membranes were incubated with the indicated primary antibodies at 4°C overnight and appropriate HRP‐linked secondary antibodies. The protein bands were developed by using ECL detection reagent (Beyotime).

### 2.9 Wound healing assay

KYSE150 and KYSE30 cells were placed into 6‐well plates (Corning) to allow the formation of confluence cell monolayer, and then the wound healing assay was performed as previously described.^25^

### 2.10 Cell migration and invasion assays

The migration and invasion of ESCC cells were carried out by using transwell chambers (8 μm, Corning) as our previously described.^5^

### 2.11 Transfection of plasmids and short hairpin RNAs (shRNAs)

pCMV‐Flag‐His‐puro‐PAK1 and pCMV‐Flag‐His‐puro (empty vector) plasmids were purchased from Transheep. For PAK1 overexpression, two μg of the indicated plasmids was transduced into KYSE150 cells by using the Lipofectamine 2000 reagent (Invitrogen, Thermo Fisher Scientific, Inc) following the manufacturer's instructions.^5^ Cells were selected with 1.5 μg/mL puromycin (Sigma‐Aldrich). After selection for 4 weeks, stable colonies were picked up and further expanded.

Specific shRNAs targeting PAK1 and shNC (pLKO.1‐puro‐No‐target shRNA) were obtained from Sigma‐Aldrich. The indicated target sense sequences were as follows: shNC (No target shRNA), 5′‐GCGATAGCGCTAATAATTT‐3′; shPAK1#1, 5′‐ATTCGAACCAGGTCATTCA‐3′; shPAK1#2, 5′‐CTAAACCATGGTTCTAAAC‐3′. Lentiviruses production and subsequently transfection were carried out as described previously.^5^ The transduced cells were selected by 1.5 μg/mL puromycin (Sigma‐Aldrich) treatment for at least 2 weeks. Immunoblotting analysis was conducted to determine the efficiency of overexpression or knockdown.

### 2.12 Mouse models of tumorigenesis and lung metastasis

Male nude BALB/c mice (5 to 6 weeks of age), purchased from Beijing Vital River Laboratory Animal Technology Co (Beijing, China), were housed in barrier system with a lighting cycle of 12 hours light/darkness, 40%—50% humidity and temperature (20 ± 2°C). All experimental procedures were approved by the Henan University Institutional Animal Care and Use Committee.

For tumour growth experiment, the nude mice were subcutaneously inoculated with KYSE150 cells (5 × 10^6^ cells) into the left dorsal flank per mouse.^22^ Tumours were examined with calipers every 2 days to measure the length and width, and tumour volume = *L* × *W* ^2^ × 0.5. Six days latter, when tumour volume was about 100 mm^3^, the mice were randomly divided into two groups and intraperitoneally injected with ivermectin (5 mg/kg) or vehicle (10% ricinus oil), respectively.^17^ Fourteen days latter, the animals were killed, and tumours were weighed and kept at −80°C or fixed in formaldehyde.

For the lung metastasis model, the BALB/c nude mice were intravenously injected with 1 × 10^6^ KYSE150 cells (100 μL PBS) via lateral tail vein^26^, ^27^ and were randomly divided into two groups. Twenty‐four hours latter, the mice treated as the above described. All of the mice were killed 8 weeks after injection. Lungs were fixed in Bouin's solution for 24 hours, and then embedded in paraffin by using the routine method. The number of metastatic colonies in lung of each mouse was counted, and histological evidence of the tumour phenotype was examined by H&E staining.

### 2.13 Immunohistochemistry (IHC) and H&E staining

After fixation in formalin for 24 hours, the tumour tissues were treated and embedded following the routine method. The IHC and H&E staining were performed as our previously described.^5^

### 2.14 Statistical analysis

All quantitative results were obtained from experiments conducted at least three times and expressed as mean ± standard deviation (SD). GraphPad Prism version 7.0 (GraphPad software) was used to conduct the statistical analyses. Student's *t* test was employed to compare the differences between two groups; whereas multiples groups were compared by using one‐way ANOVA with Tukey's post hoc test. *P* < .05 was considered statistically significant.

## 3 RESULTS

### 3.1 Ivermectin inhibits ESCC cell growth

**Figure 1:**
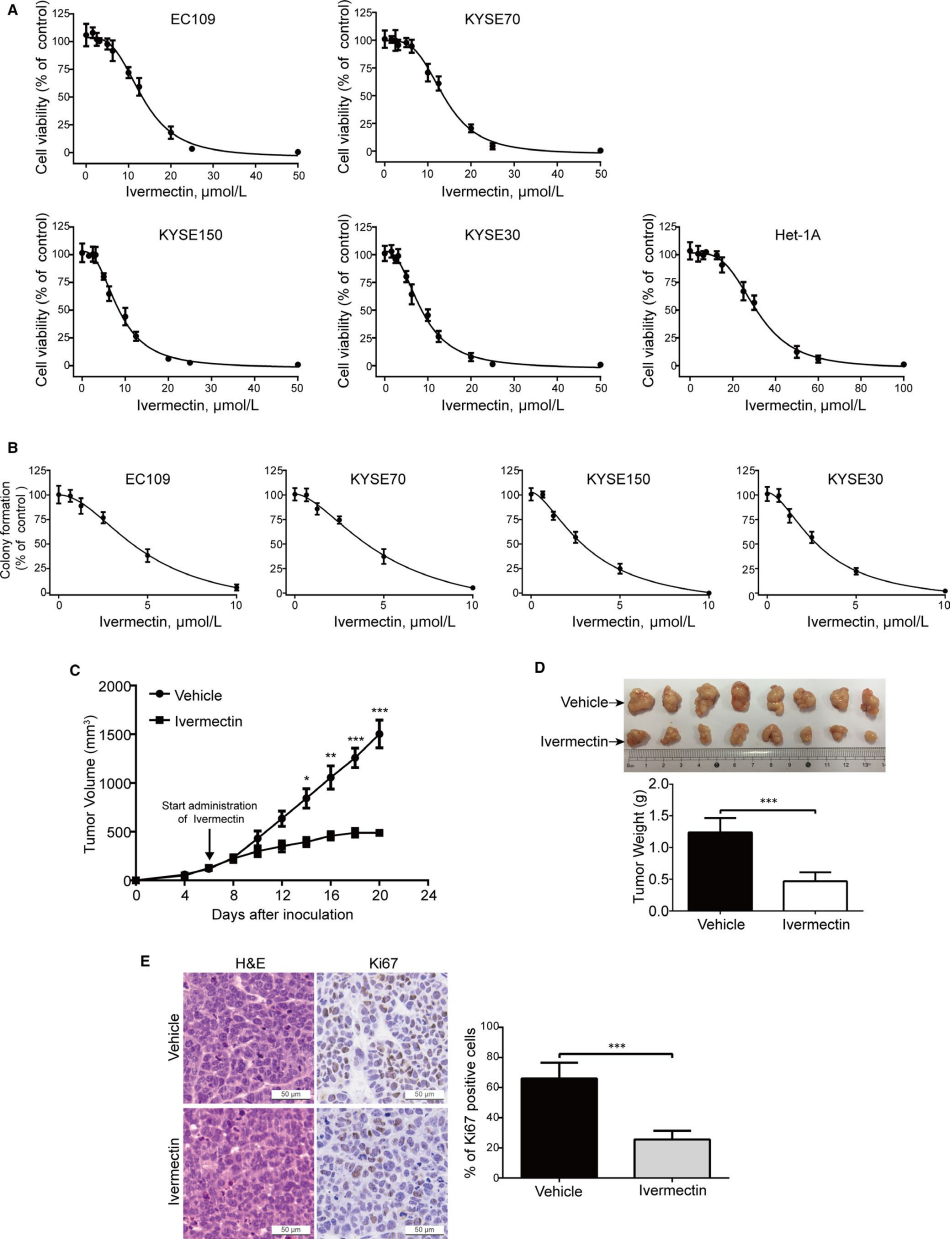
Ivermectin inhibits the growth of ESCC cells in vitro and in vivo. (A) Ivermectin inhibited ESCC cell viability. ESCC cells (KYSE70, EC109, KYSE150 and KYSE30) and one immortalized oesophageal epithelial cell line (Het‐1A) were exposed to escalating concentrations of ivermectin for 72 h, and the cell viability was determined by MTT assay. **(**B) ESCC cells were seeded in drug‐free soft agar culture and incubated for 14 days, colonies were counted. Clonogenicity was expressed by normalizing to the control group. (C‐E) Nude mice bearing palpable KYSE150 xenografted tumours were treated with vehicle or ivermectin daily. The tumour volumes measured at indicated time‐points versus time were plotted (C). Error bars, SD. n = 8 per group. Photograph of isolated tumours derived from vehicle or ivermectin‐treated mice (D, *top*). Tumour weights at time of sacrifice (D, *bottom*). (E) IHC analysis of Ki67 in xenograft tissues from mice. H&E‐stained serial sections of the same xenografts are presented. Scale bar: 50 μm. *P < .05, **P < .01, ***P < .001, by Student's *t* test

To ascertain the anti‐proliferative effect of ivermectin on ESCC cells, MTT assay was performed to measure the cell viability of four ESCC cell lines (EC109, KYSE30, KYSE70 and KYSE150) and one immortalized oesophageal epithelial cell line (Het‐1A) following ivermectin treatment for 72 hours. As shown in Figure 1A, the cell viability of four ESCC cell lines (EC109, KYSE70, KYSE150 and KYSE30) was markedly inhibited by ivermectin, with IC_50_ values of 13.35, 13.86, 8.49 and 8.56 μmol/L, respectively. While the IC_50_ value in Het‐1A cells was much higher than those in ESCC cells, with IC_50_ values of 31.21 μmol/L. Moreover, soft agar assay also showed that the clonogenicity of ESCC cells was effectively suppressed in a concentration‐dependent manner upon ivermectin treatment (Figure 1B).

To determine the inhibitory effect of ivermectin on ESCC cell growth in vivo, we employed an xenografted model by subcutaneously injecting KYSE150 cells into *nu/nu* BALB/c nude mice. As illustrated in Figure 1C, ivermectin‐treated xenografts grew at a slower rate than those of the control group. Similarly, the size of ivermectin‐treated tumours was much smaller than that of control group (Figure 1D). Consistently, tumour weight in mice treated with ivermectin was reduced compared with that of the vehicle‐treated group (Figure 1D). To determine whether ivermectin can change the proliferation status of ESCC cells in vivo, xenografts were stained for the proliferative marker Ki67 by IHC analysis. The results showed that xenografts derived from ivermectin‐treated mice displayed much weaker Ki67 staining and a smaller proportion of Ki67 positive cells than that of vehicle‐treated mice (Figure 1E).

Overall, these results demonstrated that ivermectin effectively suppresses the growth of ESCC cells in vitro and in vivo.

### 3.2 Ivermectin elicits ESCC cell apoptosis through the mitochondrial signalling pathway

**Figure 2:**
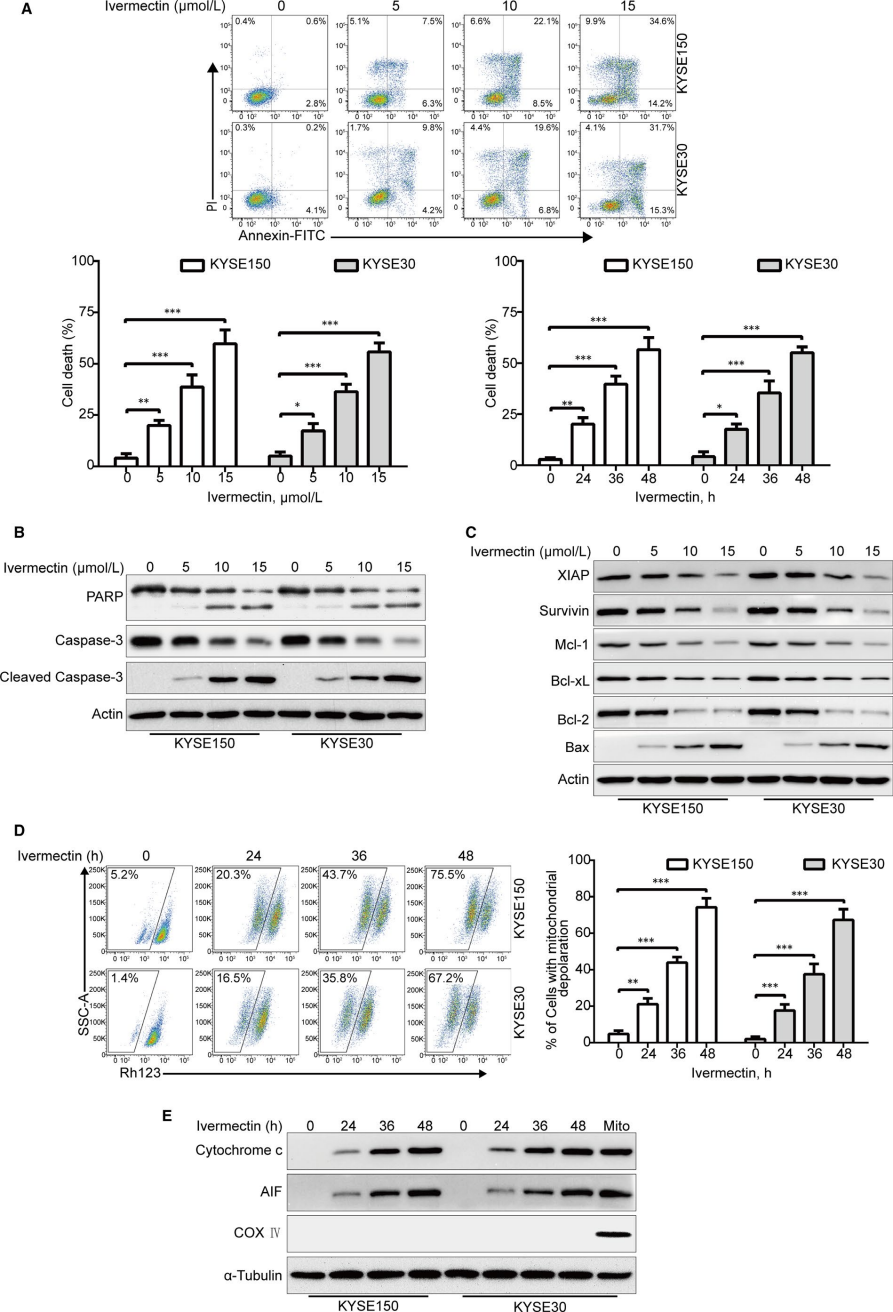
Ivermectin induces apoptosis via mitochondrial pathway in ESCC cells. (A) KYSE150 and KYSE30 cells were exposed to increasing concentrations of ivermectin for 48 h, or to 15 μmol/L ivermectin for different time periods, and the apoptotic cells were assessed by flow cytometry after dual‐staining with Annexin V‐FITC and PI *Top*, Representative flow cytometry dot plots are shown; *Bottom*, Quantitative analysis of dead cells from three independent experiments. Dead cells were the sum of cells with single‐ or dual‐stained by Annexin V or PI. *P < .05, **P < .01, ***P < .001, one‐way ANOVA with post hoc intergroup comparison by the Tukey's test. (B, C) KYSE150 and KYSE30 cells were exposed to various concentrations of ivermectin for 48 h, immunoblotting analysis was performed to detect the apoptotic proteins. Actin served as loading control. (D) KYSE150 and KYSE30 cells treated with or without 5 μmol/L ivermectin for increasing durations, and then the mitochondrial membrane potential was assessed by flow cytometry after Rh123 staining. *Right*: Results from five independent experiments. *P < .05, **P < .01, ***P < .001, one‐way ANOVA with post hoc intergroup comparison by the Tukey's test. (E) Ivermectin induced the release of Cytochrome c and AIF into cytosol in ESCC cells. Levels of Cytochrome c and AIF in the cytosolic extracts prepared with digitonin buffer were examined by western blotting analysis. COX Ⅳ served as a mitochondrial indicator to rule out contamination of cytosolic fractions from mitochondria. α‐Tubulin served as internal control

To explore whether ivermectin induces ESCC cell apoptosis, KYSE150 and KYSE30 cells were pre‐treated with various doses of ivermectin, cells were harvested, stained with PI and Annexin V‐FITC, and then assessed by flow cytometry. As shown in Figure 2A, ivermectin treatment led to remarkable apoptotic cell death in a dose‐ and time‐dependent manner in both KYSE150 and KYSE30 cells. Consistently, Western blotting analysis showed a concentration‐dependent increase of the cleavage of PARP and activation of Caspase‐3, the expression of pro‐Caspase‐3 was constructively down‐regulated, further confirming that ivermectin‐triggered apoptosis in ESCC cells (Figure 2B).

In order to elucidate the underlying molecular mechanism of ivermectin‐induced apoptosis, immunoblotting analysis was employed to detect the effect of ivermectin on apoptosis‐related proteins. As shown in Figure 2C, upon treatment with ivermectin, the expression of anti‐apoptotic proteins including IAP family proteins (XIAP and Survivin) and Bcl‐2 family members (Bcl‐xL, Bcl‐2 and Mcl‐1) were greatly inhibited; Conversely, the pro‐apoptotic protein Bax was remarkably up‐regulated (Figure 2C). To further explore the underlying mechanism of ivermectin‐mediated cell apoptosis, KYSE150 and KYSE30 cells were incubated with 5 μmol/L ivermectin for different time periods, and then stained with rhodamine 123 (Rh123), the MMP was measured by flow cytometry analysis. As illustrated in Figure 2D, the MMP was significantly down‐regulated in a time‐dependent fashion, which was in line with other apoptotic indices including PARP cleavage and active Caspase‐3 (Figure 2B). Next, we explored whether ivermectin triggers the release of Cytochrome c into the cytosol. Immunoblotting analysis showed that ivermectin treatment led to a time‐dependent up‐regulation of AIF and Cytochrome c in cytoplasm (Figure 2E). Altogether, these findings indicate that ivermectin induces ESCC cell apoptosis via the mitochondrial signalling pathway.

### 3.3 Ivermectin suppresses ESCC cell migration and invasion

**Figure 3:**
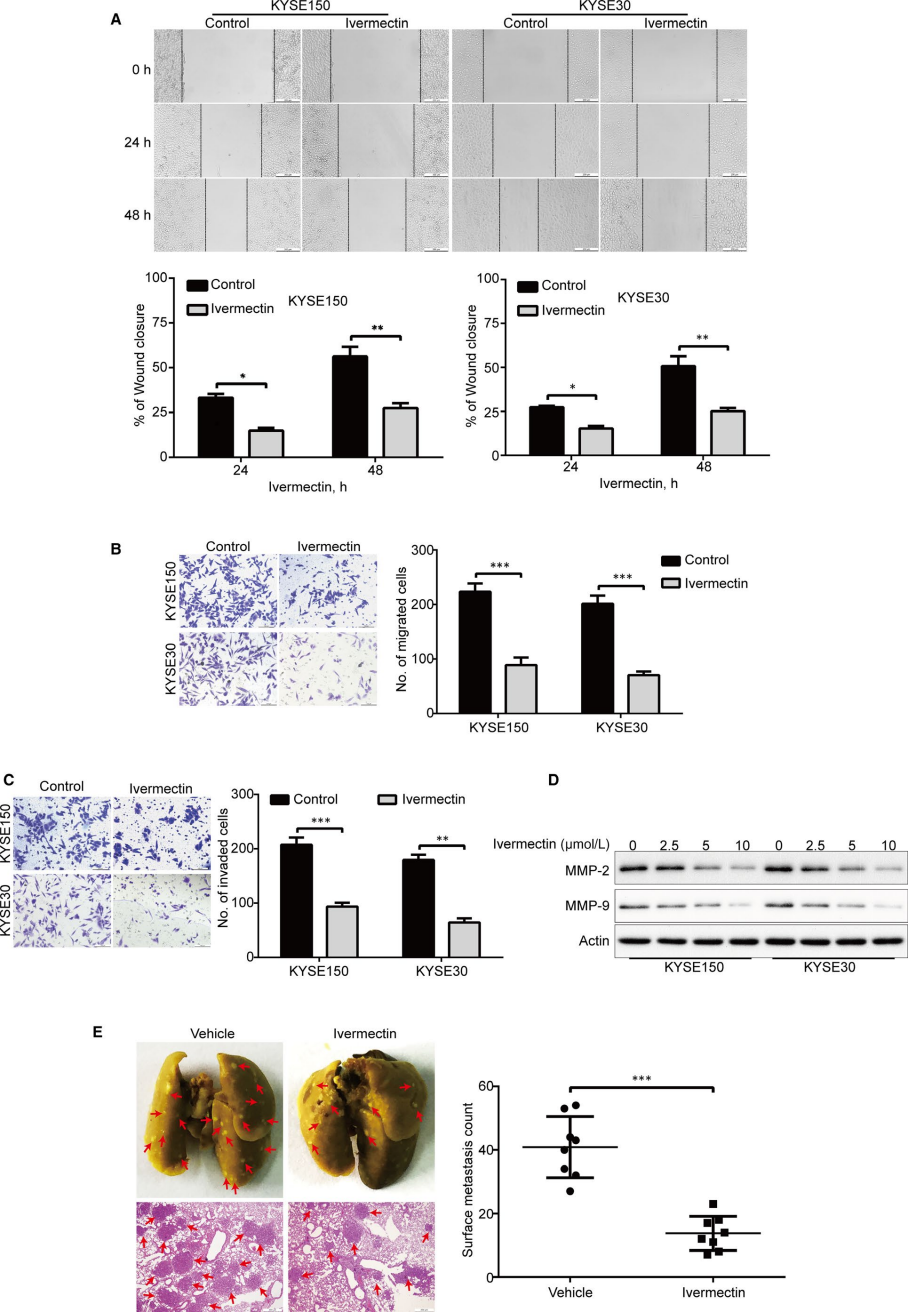
Ivermectin suppresses migration, invasion and metastasis of ESCC cells. (A) KYSE150 and KYSE30 cells treated with or without 2.5 μmol/L ivermectin were subjected to wound healing assay. *Top*: Representative images were recorded at 0 h, 24 h, 48 h time‐point. *Bottom*: Quantitative analysis of the relative breadth of the wound from five independent experiments. The wound breadth was normalized to the initial time‐point (0 h). Columns and error bars represent mean ± SD (n = 5 per group). (B, C) KYSE150 and KYSE30 cells were pre‐treated with 2.5 μmol/L ivermectin for 48 h, and then subjected to transwell migration (B) and invasion (C) assays. *Left*: Representative images; *Right*: Quantitative analysis of five independent experiments. Scale bar: 100 μm. Mean; error bar, SD. (D) Western blotting analysis of whole cell lysates of KYSE150 and KYSE30 cells that were pre‐treated with different concentrations of ivermectin for 48 h. *P < .05, **P < .01, ***P < .001, by Student's *t* test. (E) KYSE150 cells were intravenously injected into nude mice via the lateral tail vein. *Left*: Representative images of lungs (upper panel) and lung sections stained with H&E (lower panel) harvested 8 weeks post‐injection (arrows indicate the metastatic tumours). *Right*: Surface metastatic nodules in the lungs were counted. Data represent mean ± SD (n = 8 per group). Scale bar, 200 μm. ***P < .001 by Student's *t* test

We next explored whether ivermectin suppresses the migration and invasion, two important characteristic features involved in cancer metastasis including ESCC. In wound healing assay, upon treatment with 2.5 μmol/L ivermectin for 24 hours, the migration of KYSE150 and KYSE30 cells that was dramatically suppressed, the inhibition was further enhanced after incubation with ivermectin for 48 hours (Figure 3A). Consistent with these results, transwell migration assay revealed that the migrative ability was greatly attenuated by ivermectin at the concentration of 2.5 μmol/L in both tested ESCC cell lines (Figure 3B). We also explore whether ivermectin has a inhibitory effect on ESCC cell invasion by using transwell invasion assay. Interestingly, the data showed that ivermectin strongly diminished the invasion of KYSE150 and KYSE30 cells, compared with corresponding control groups (Figure 3C). Consistently, the protein levels of MMP‐9 and MMP‐2, two important family members of matrix metalloproteinases (MMPs), were greatly down‐regulated in a dose‐dependent manner in both KYSE150 and KYSE30 cells (Figure 3D). Collectively, these data suggest that ivermectin effectively suppresses the migration and invasion of ESCC cells.

### 3.4 Ivermectin diminishes ESCC metastasis in nude mice

Given that ivermectin could obviously attenuate the migration and invasive abilities of ESCC cells in vitro, we then investigated whether ivermectin inhibits the tumour metastasis in vivo. A lung metastasis mouse model was performed by intravenously injecting KYSE150 cells into nude mice via lateral tail veins and given ivermectin treatment for 14 days. As illustrated in Figure 3E, ivermectin substantially decreased the ability of lung metastasis, as indicated by diminished number of lung metastases in the lung of each animal, compared to the control group. Moreover, the results from H&E staining revealed an obviously reduced in the number of metastatic nudes and tumour size in the lungs of ivermectin‐treated mice, compared to that of the control group (Figure 3E). Altogether, these in vivo results indicate that ivermectin effectively diminishes the tumour metastasis of ESCC.

### 3.5 Ivermectin inhibits ESCC cell growth, migration and invasion via down‐regulation of PAK1

**Figure 4:**
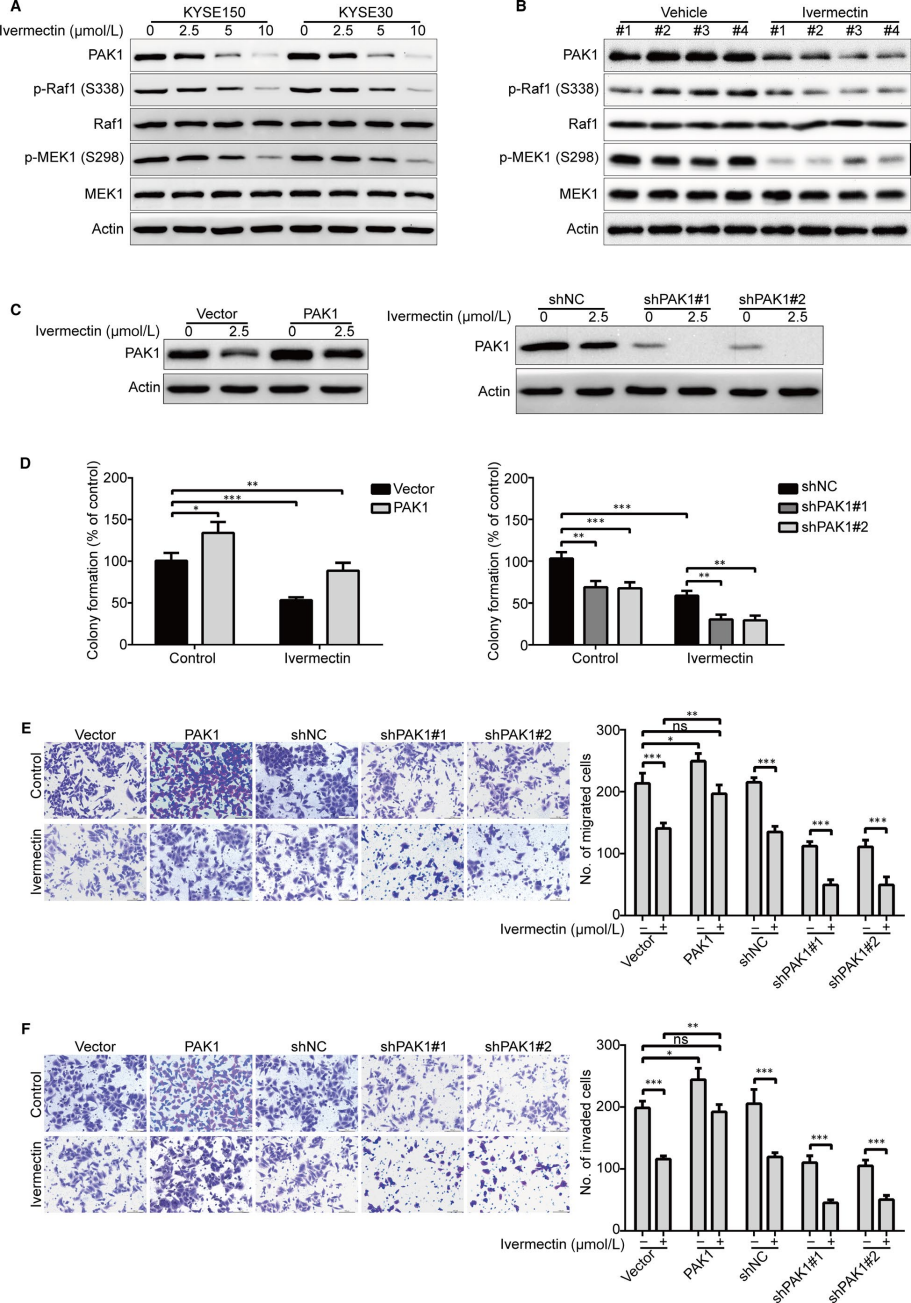
Ivermectin suppresses cell growth, migration and invasion through down‐regulation of PAK1 in ESCC cells. (A) KYSE150 and KYSE30 cells were treated with the indicated concentrations of ivermectin for 48 h, and then the expression of PAK1 and its downstream targets was analysed by Western blotting. (B) Western blotting analysis of the whole lysates derived from xenografted tumour tissues of vehicle‐ or ivermectin‐treated mice. (C‐F) KYSE150 cells stably transduced with either PAK1 plasmids or lentivirus PAK1‐shRNA constructs were treated with or without ivermectin for 48 h. The whole cell lysates were used to measure the expression of PAK1 by immunoblotting (C). ESCC cells were harvested and subjected to soft agar (D), transwell migration (E) and invasion (F) assays. ns: no significant difference; *P < .05, **P < .01, ***P < .001, one‐way ANOVA with post hoc intergroup comparison by the Tukey's test

Increasing evidence has demonstrated that high expression of PAK1 was observed in various cancers including ESCC, and PAK1 played an crucial role in regulating tumour growth and metastasis.^5^ Therefore, we determine whether PAK1 is inhibited in ivermectin‐treated ESCC cells. KYSE150 and KYSE30 cells were pre‐treated with escalating concentrations of ivermectin; cells were harvested and underwent Western blotting analysis of PAK1. As illustrated in Figure 4A, ivermectin greatly inhibited the expression of PAK1 in a dose‐dependent manner. Interestingly, the phosphorylation of Raf1 (S338) and MEK1 (S298), two validated downstream substrates of PAK1,^28^, ^29^ was also obviously suppressed by ivermectin (Figure 4B). Consistently, the protein levels of PAK1, p‐Raf1 (S338) and p‐MEK1 (S298) were greatly diminished in the tumour xenografts derived from ivermectin‐treated mice, compared to the vehicle‐treated controls (Figure 4B).

In order to further explore whether PAK1 is involved in ivermectin‐mediated inhibitory effect on ESCC cells. KYSE150 cells were stably overexpressed PAK1, followed by treatment without or with ivermectin for forty‐eight hours, and then subjected to soft agar, transwell migration and invasion experiments. The results showed that PAK1 expression was much higher in KYSE150‐PAK1 cells than those cells transfected with empty vector (Figure 4C, *left* panel). In soft agar assay, ectopic overexpression of PAK1 not only increased the colony‐forming abilities, but also significantly attenuated the inhibition of colony formation induced by ivermectin (Figure 4D, *left* panel). Additionally, overexpression of PAK1 nearly completely rescued the inhibitory effects of ivermectin on the migration (Figure 4E) and invasion (Figure 4F) in KYSE150 cells. Vice versa, knockdown of PAK1 by lentiviral shRNA (Figure 4C, *right* panel) significantly suppressed the colony formation (Figure 4D, *right* panel), migration (Figure 4E) and invasion (Figure 4F), similar to the results mediated by ivermectin. More importantly, silencing PAK1 enhanced the abrogation of colony formation (Figure 4D, *right* panel), migration (Figure 4E) and invasion (Figure 4F) induced by ivermectin. These data confirmed that PAK1 is involved in ivermectin‐mediated inhibitory effects on ESCC cells.

### 3.6 Ivermectin promotes degradation of PAK1 through the proteasome‐dependent pathway

**Figure 5:**
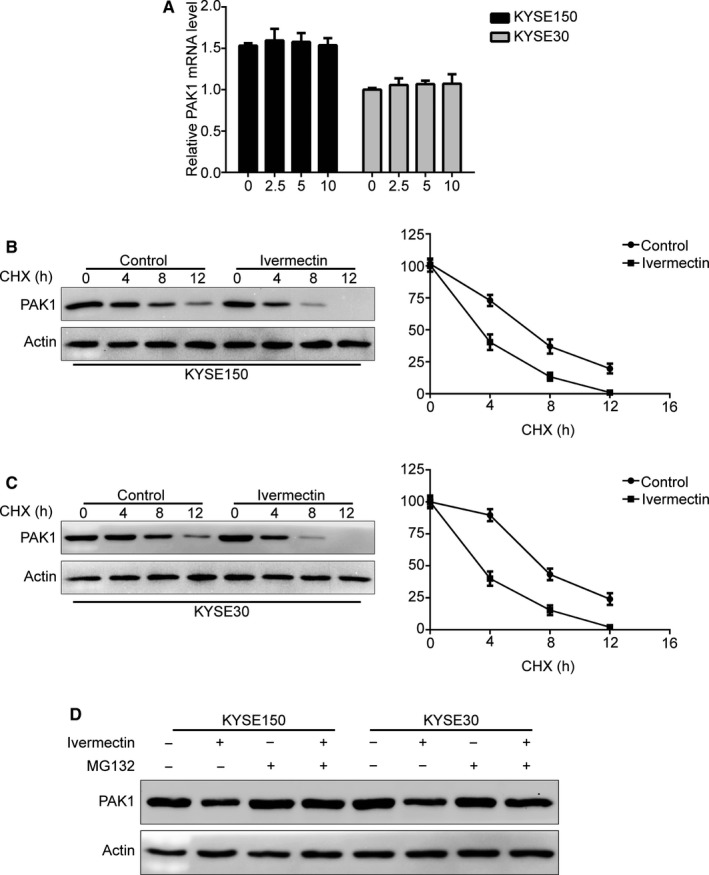
Ivermectin promotes ubiquitin degradation of PAK1. (A) KYSE150 and KYSE30 cells were treated with the indicated concentrations of ivermectin for 48 h, and then the PAK1 mRNA expression was evaluated by qRT‐PCR. (B, C) KYSE150 (B) and KYSE30 (C) cells were treated with 2.5 μmol/L ivermectin for 36 h, followed by treatment with cycloheximide (CHX, 50 μmol/L) for another 0‐12 h. The whole cell lysates were extracted for Western blotting with anti‐PAK1 antibody (*left*). Triplicate experiments were performed for statistical analysis (*right*). (D) Immunoblotting analysis of PAK1 expression in ESCC cells pre‐treated with MG132 (5 μmol/L) for 2 h, and then with or without 2.5 μmol/L ivermectin treatment for 48 h. *P < .05, **P < .01, ***P < .001, by Student's *t* test

In order to elucidate the underlying molecular mechanism by which ivermectin down‐regulated the protein level of PAK1, qRT‐PCR analysis was conducted to quantify PAK1 mRNA expression. However, there was no obvious difference between ivermectin‐treated ESCC cells and control groups in regulation of PAK1 mRNA level (Figure 5A), suggesting that ivermectin‐mediated down‐regulation of PAK1 might not be at the transcription level. We next investigated whether the degradation of PAK1 induced by ivermectin is at the post‐translational level by using cycloheximide (CHX), a protein synthesis inhibitor. KYSE150 and KYSE30 cells were pre‐treated with 2.5 μmol/L ivermectin for 36 hours, followed by additional treatment without or with CHX (50 μg/mL) for 0‐12 hours. Immunoblotting results showed that an impaired turnover rate in PAK1 protein in ivermectin‐treated KYSE150 cells was observed (Figure 5B). Similarly, time chase experiments also showed that increased degradation of PAK1 level in ivermectin‐treated KYSE30 cells, compared to the controls (Figure 5C). We next explored whether the degradation of PAK1 induced by ivermectin was through the proteasome‐dependent signalling pathway. Interestingly, upon exposure to MG132 (a specific proteasome inhibitor), the expression of PAK1 protein was drastically rescued compared with ivermectin alone treated groups in both tested cell lines of ESCC (Figure 5D), suggesting ivermectin‐triggered degradation of PAK1 is via the proteasome‐dependent signalling pathway in ESCC cells.

### 3.7 Ivermectin synergizes with CDDP and 5‐FU to induce apoptosis of ESCC cells

**Figure 6:**
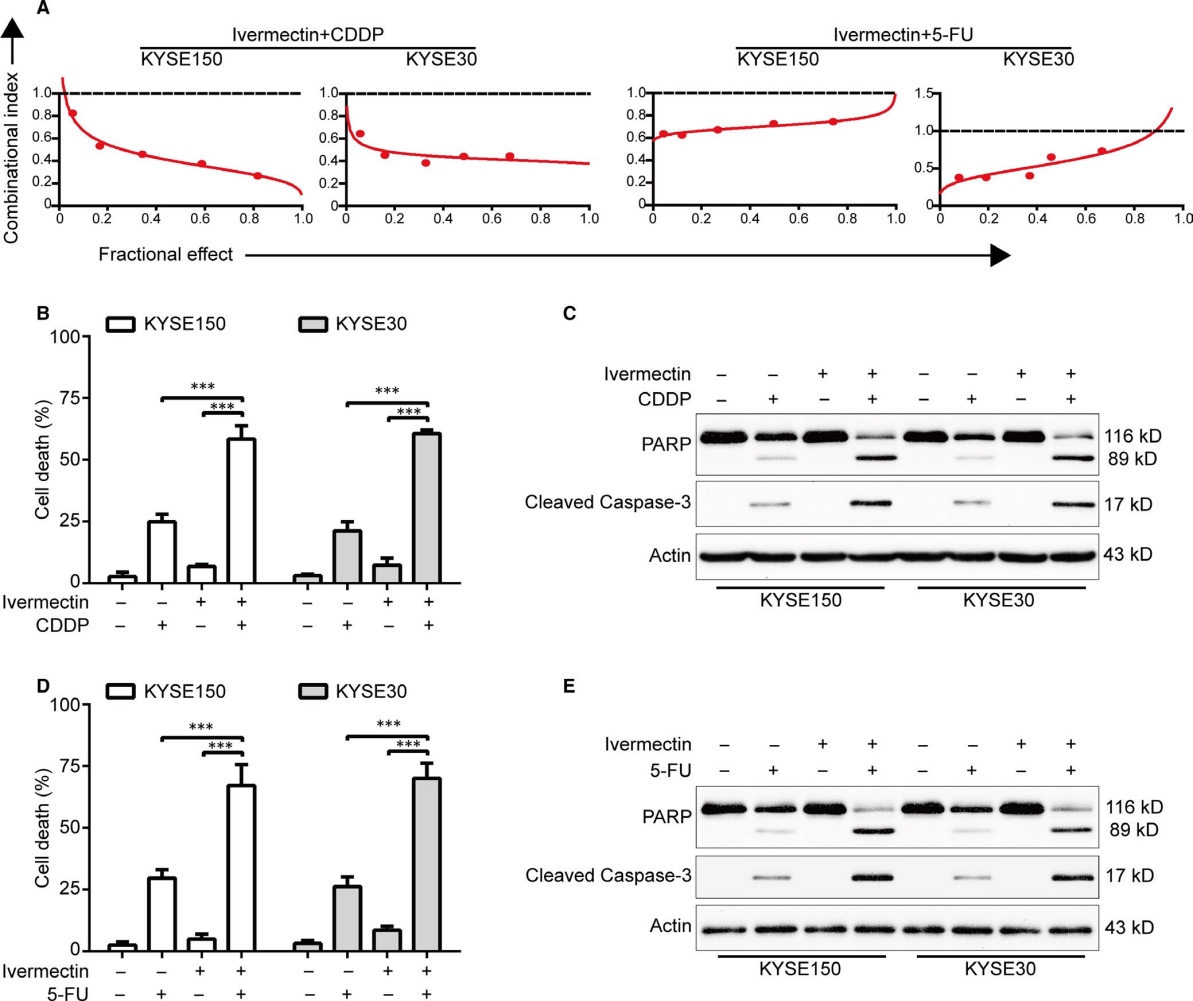
Ivermectin is synergistic with CDDP and 5‐FU. (A) KYSE150 and KYSE30 cells were exposed to a serially diluted mixture at a fixed ratio of ivermectin and CDDP or 5‐FU for 72 h, cell viability was measured by MTT assay. The median‐effect method of Chou and Talalay was used to assess the synergistic effect. The combination index (CI) was the ratio of the combination dose to the sum of the single‐agent doses at an isoeffective level. CI < 1 indicates synergy and CI = 1 indicates additive. (B) and (D) KYSE150 and KYSE30 cells were exposed to ivermectin (2.5 μmol/L) with combination of CDDP (12.5 μmol/L) or 5‐FU (200 μmol/L) for 48 h, and then examined with a haemocytometer by trypan blue exclusion assay (n = 6 per group). Column, mean; Error bar, SD. ***, *P* < .001, one‐way ANOVA with post hoc intergroup comparison with Tukey's test. (C) and (E) Western blotting analysis of the cleaved PARP and Caspase‐3. – indicates the absence and + indicates the presence of the drugs

Mounting evidence has demonstrated that novel therapeutic agents in combination with conventional chemotherapeutic drugs are more effective than each kind of agents alone to improve the life quality of patients with ESCC.^30^, ^31^ CDDP and 5‐FU are two front‐line chemotherapeutic drugs in use for ESCC.^30^ We therefore examined the efficiency of ivermectin combined with CDDP or 5‐FU in KYSE150 and KYSE30 cells. Cells were treated with ivermectin alone, or in combination with the indicated chemotherapeutic agent, three days latter, cell viability was performed by MTT assay and the combined effect was assessed according to the combination index (CI). As shown in Figure 6A, there was a synergistic effect between ivermectin and CDDP or 5‐FU in reducing cell viability of the two tested ESCC cells. With another experiment, ESCC cells were exposed to 2.5 μmol/L ivermectin with combination of 12.5 μmol/L CDDP or 5‐FU (200 μmol/L) for 48 hours, the percentage of dead cancer cells was detected by using trypan blue exclusion assay. The results showed that ivermectin alone displayed no obvious toxicity to KYSE150 and KYSE30 cells, and either CDDP or 5‐FU just triggered minimal cell death. However, the combination of ivermectin and CDDP or 5‐FU led to a remarkable increase in the ratio of cell death (Figure 6B,D). Consistently, immunoblotting analysis also confirmed that ivermectin enhanced the apoptosis‐induced by CDDP, as demonstrated by Caspase‐3 activation and cleavage of PARP in KYSE150 and KYSE30 cells (Figure 6C). Similar results were observed when ivermectin combined with 5‐FU (Figure 6D). These findings suggest that the combinational regimen of ivermectin and CDDP or 5‐FU provides a novel therapeutic approach for patients with ESCC.

## 4 DISCUSSION

In this study, we found that ivermectin was effective in suppressing ESCC cell growth, migration and invasion. More importantly, our data also showed that ivermectin had a high antitumour activity against tumour growth and metastasis in nude mouse models. Additionally, ivermectin‐induced apoptosis and enhanced the cell growth inhibition induced by CDDP or 5‐FU. To our knowledge, this is the first study to investigate the antitumour activity of ivermectin against ESCC in vitro and in vivo.

Disclosing new uses for old drugs is often much more economical and faster than developing new drugs from scratch.^32^, ^33^ In this study, we discovered that ivermectin which has been approved to treat human diseases in the USA and other countries for over 30 years, showed potent antitumour activity against ESCC both in vitro and in nude mouse models. In line with our results, a few studies have demonstrated that ivermectin displayed an potent antitumour activity against diverse malignant tumours such as glioma, breast cancer, non‐small cell lung cancer and colon cancer in vitro and in nude mice.^14^, ^17^, ^20^

In this study, we also noted that ivermectin drastically induced apoptosis in both tested ESCC cells based on the following facts: (a) Ivermectin treatment resulted in increased proportions of annexin V binding cells in a concentration‐ and time‐dependent fashion; (b) Specific activation of Caspase‐3 and PAPP were observed upon ivermectin treatment; (c) The levels of pro‐survival proteins including XIAP, Survivin, Bcl‐2, Bcl‐xL and Mcl‐1 were down‐regulated, while the pro‐apoptotic protein Bax was up‐regulated in ivermectin‐treated ESCC cells. (d) Ivermectin treatment led to a time‐dependent down‐regulation of the mitochondrial transmembrane potential and release of AIF and Cytochrome c from mitochondrial into the cytosol. Consistent with our findings, Melotti A and colleagues found that ivermectin treatment led to a sevenfold increase in the number of activated Caspase‐3 positive cells in both primary and cell lines of colon cancer.^14^ In addition, ivermectin also induced epithelial ovarian cancer (EOC) cell apoptosis and promoted the protein levels of cleaved PARP, Bax, p27 and p21.^18^ Whereas, Dou et al reported that ivermectin did not induce cell apoptosis, but significantly stimulated cytostatic autophagy in breast cancer cells.^17^ Taken together, these findings suggest that whether ivermectin induces cell apoptosis may depend on the tumour context.

Tumour metastasis is one of the major causes that lead to treatment failure in ESCC patients. Multistep processes including cancer cell adhesion, migration and invasion into adjacent vasculature or tissues are involved in tumour metastasis. In this report, by using scratch and transwell migration assays, ivermectin displayed highly inhibitory activity against the migration of ESCC cells. Moreover, we also found that ivermectin effectively suppressed ESCC cell invasion. MMP‐9 and MMP‐2 are two key family members of MMPs that are able to degrade the basement membranes and extracellular matrix, and thus facilitate invasion and metastasis of malignant tumour cells into other organs or tissues.^34^ Intriguingly, the protein levels of MMP‐2 and MMP‐9 were obviously suppressed by ivermectin. More importantly, we also found ivermectin greatly suppressed the lung metastasis in nude mice models. Consistently, Kwon *et al* reported that ivermectin treatment suppressed cell invasion and up‐regulated E‐cadherin expression in triple‐negative breast cancer (TNBC) cells.^35^ Similarly, upon treatment with ivermectin, the invasive ability of glioma cells was also potentially inhibited.^20^ Altogether, our data suggest that ivermectin is a promising therapeutic agent for malignant cancer patients with metastasis.

PAK1, a member of P21‐activated kinases (PAKs), is an important serine/threonine‐protein kinase that can be activated by the small Rho family of GTPases (Rac1 and Cdc42).^36^ Accumulating evidence indicates that PAK1 is overexpression in diverse malignant tumours such as non‐small cell lung cancer, breast cancer, hepatocellular carcinoma and pancreatic cancer, and high expression of PAK1 is positively associated with high risk of tumour recurrence and metastasis, and poor survival.^37^, ^38^, ^39^, ^40^ Consistently, we recently found that PAK1 is highly expressed in ESCC, and targeting PAK1 by genetic or pharmacological inhibition drastically suppressed cell growth, migration, invasion and metastasis.^5^ All these findings indicate PAK1 is a potential therapeutic target for diverse cancers including ESCC. In the present study, we demonstrated that ivermectin suppressed ESCC cell growth, migration and invasion through inducing PAK1 degradation via the proteasome‐dependent signalling pathway, and the conclusion is based on the following effects: (a) Ivermectin greatly inhibited the expression of PAK1 protein in both tested ESCC cell lines and tumour xenografts, but had no obvious effect on PAK1 mRNA level, indicating that ivermectin‐mediated down‐regulation of PAK1 might not be at the transcription level. (b) Silencing PAK1 obviously enhanced the inhibitory effects of ivermectin on colony formation, migration and invasion; conversely, enforced expression of PAK1 resulted in a completely opposing phenomenon; (c) Time chase experiments in the presence of CHX showed that an increased degradation rate of PAK1 level was noted in ivermectin‐treated ESCC cells, compared to that of the controls, suggesting ivermectin down‐regulated PAK1 at the post‐translational level; (d) The down‐regulation of PAK1 induced by ivermectin could be remarkably restored by the specific proteasome inhibitor MG132 in both tested cell lines of ESCC. In line with our findings, Dou *et al* reported that ivermectin could induce breast cancer cell autophagy by promoting PAK1 degradation via the ubiquitination pathway.^17^

The traditional chemotherapeutic agents CDDP and 5‐FU are commonly used chemotherapeutic agents for patients with locally advanced ESCC.^41^ However, the side effects and survival rates were remained unsatisfactory, with 25% grade 3 late toxicities, 42% acute toxicities, and the overall five‐year survival rate was only 26%.^41^, ^42^ In this study, the data showed that there was a synergistic inhibitory effect between ivermectin and CDDP or 5‐FU in reducing ESCC cell viability. Additionally, we demonstrated that ivermectin improved the sensitivity of ESCC cells to these cytotoxic drugs partially by inducing cell apoptosis, as demonstrated by specific activation of PARP and Caspase‐3. Thus, our findings suggest that ivermectin treatment might decrease the effective dose of chemotherapeutic agents as well as improve the anti‐cancer effects of these agents. Consistently, Sharmeen S *et al* found that ivermectin could synergize with daunorubicin and cytarabine to trigger cell death in leukaemia cells.^15^ In addition, Kodama et al reported that ivermectin in combination with paclitaxel displayed a stronger antitumour effect on epithelial ovarian cancer (EOC) than either drug alone both in vitro and in vivo.^18^ Altogether, these findings from us and others suggest that combination of ivermectin with the standard treatment drugs is a potential therapeutic strategy for cancer patients. Thus, future work needs to further investigate the efficiency of ivermectin in combination with traditional chemotherapeutic agents in mouse models and clinical trials of ESCC.

In summary, our study demonstrated that ivermectin effectively suppressed ESCC cell growth, migration and invasion through blocking the PAK1 signalling. We also found that ivermectin displayed significant antitumour effects on tumorigenesis and lung metastasis in xenografted mouse models, and improved the sensitivity to the first‐line chemotherapeutic drugs including CDDP or 5‐FU. Given that ivermectin has been approved by FDA for treatment of human diseases, our data warrant a clinical trial of ivermectin for treatment of ESCC patients, even those with metastasis or chemotherapeutic resistance.

## CONFLICT OF INTEREST

The authors declare that there are no conflicts of interest.

## AUTHOR CONTRIBUTION

Liang Chen designed, performed experiments, analysed data and drafted the manuscript; Shuning Bi and Qiuren Wei performed experiments; Zhijun Zhao guided research and analysed data; Chaojie Wang and Songqiang Xie designed, guided research and wrote the manuscript. All authors read and approved the final manuscript.

## Data Availability

The data used to support the findings in this study are available upon reasonable request from the corresponding authors.

## References

[1] Torre LA , Bray F , Siegel RL , Ferlay J , Lortet‐Tieulent J , Jemal A . Global cancer statistics, 2012. CA Cancer J Clin. 2015;65:87‐108. 25651787 10.3322/caac.21262

[2] Murphy G , McCormack V , Abedi‐Ardekani B , et al. International cancer seminars: a focus on esophageal squamous cell carcinoma. Ann Oncol. 2017;28:2086‐2093. 28911061 10.1093/annonc/mdx279 PMC5834011

[3] Arnold M , Soerjomataram I , Ferlay J , Forman D . Global incidence of oesophageal cancer by histological subtype in 2012. Gut. 2015;64:381‐387. 25320104 10.1136/gutjnl-2014-308124

[4] Rustgi AK , El‐Serag HB . Esophageal carcinoma. N Engl J Med. 2014;371:2499‐2509. 25539106 10.1056/NEJMra1314530

[5] Chen L , Bi S , Hou J , Zhao Z , Wang C , Xie S . Targeting p21‐activated kinase 1 inhibits growth and metastasis via Raf1/MEK1/ERK signaling in esophageal squamous cell carcinoma cells. Cell Commun Signal. 2019;17:31. 30971268 10.1186/s12964-019-0343-5 PMC6458688

[6] Cheng X , Wei L , Huang X , et al. Solute carrier family 39 member 6 gene promotes aggressiveness of esophageal carcinoma cells by increasing intracellular levels of zinc, activating phosphatidylinositol 3‐kinase signaling, and up‐regulating genes that regulate metastasis. Gastroenterology. 2017;152(1985–97):e12. 10.1053/j.gastro.2017.02.006 28209530

[7] Lin D‐C , Hao J‐J , Nagata Y , et al. Genomic and molecular characterization of esophageal squamous cell carcinoma. Nat Genet. 2014;46:467‐473. 24686850 10.1038/ng.2935 PMC4070589

[8] Kass IS , Wang CC , Walrond JP , Stretton AO . Avermectin B1a, a paralyzing anthelmintic that affects interneurons and inhibitory motoneurons in Ascaris. Proc Natl Acad Sci USA. 1980;77:6211‐6215. 6255481 10.1073/pnas.77.10.6211 PMC350245

[9] Juarez M , Schcolnik‐Cabrera A , Duenas‐Gonzalez A . The multitargeted drug ivermectin: from an antiparasitic agent to a repositioned cancer drug. Am J Cancer Res. 2018;8:317‐331. 29511601 PMC5835698

[10] Hashimoto H , Messerli SM , Sudo T , Maruta H . Ivermectin inactivates the kinase PAK1 and blocks the PAK1‐dependent growth of human ovarian cancer and NF2 tumor cell lines. Drug Discov Ther. 2009;3:243‐246. 22495656

[11] King CL , Suamani J , Sanuku N , et al. A trial of a triple‐drug treatment for lymphatic filariasis. N Engl J Med. 2018;379:1801‐1810. 30403937 10.1056/NEJMoa1706854 PMC6194477

[12] Campbell WC . Ivermectin, an antiparasitic agent. Med Res Rev. 1993;13:61‐79. 8416263 10.1002/med.2610130103

[13] Wolstenholme AJ , Rogers AT . Glutamate‐gated chloride channels and the mode of action of the avermectin/milbemycin anthelmintics. Parasitology. 2005;131(Suppl):S85‐95. 16569295 10.1017/S0031182005008218

[14] Melotti A , Mas C , Kuciak M , et al. The river blindness drug Ivermectin and related macrocyclic lactones inhibit WNT‐TCF pathway responses in human cancer. EMBO Mol Med. 2014;6:1263‐1278. 25143352 10.15252/emmm.201404084 PMC4287931

[15] Sharmeen S , Skrtic M , Sukhai MA , et al. The antiparasitic agent ivermectin induces chloride‐dependent membrane hyperpolarization and cell death in leukemia cells. Blood. 2010;116:3593‐3603. 20644115 10.1182/blood-2010-01-262675

[16] Wang J , Xu Y , Wan H , Hu J . Antibiotic ivermectin selectively induces apoptosis in chronic myeloid leukemia through inducing mitochondrial dysfunction and oxidative stress. Biochem Biophys Res Commun. 2018;497:241‐247. 29428725 10.1016/j.bbrc.2018.02.063

[17] Dou Q , Chen H‐N , Wang K , et al. Ivermectin induces cytostatic autophagy by blocking the PAK1/AKT axis in breast cancer. Cancer Res. 2016;76:4457‐4469. 27302166 10.1158/0008-5472.CAN-15-2887

[18] Kodama M , Kodama T , Newberg JY , et al. In vivo loss‐of‐function screens identify KPNB1 as a new druggable oncogene in epithelial ovarian cancer. Proc Natl Acad Sci USA. 2017;114:E7301‐E7310. 28811376 10.1073/pnas.1705441114 PMC5584430

[19] Liu Y , Fang S , Sun Q , Liu B . Anthelmintic drug ivermectin inhibits angiogenesis, growth and survival of glioblastoma through inducing mitochondrial dysfunction and oxidative stress. Biochem Biophys Res Commun. 2016;480:415‐421. 27771251 10.1016/j.bbrc.2016.10.064

[20] Yin J , Park G , Lee JE , et al. DEAD‐box RNA helicase DDX23 modulates glioma malignancy via elevating miR‐21 biogenesis. Brain. 2015;138:2553‐2570. 26121981 10.1093/brain/awv167

[21] Chou TC . Drug combination studies and their synergy quantification using the Chou‐Talalay method. Cancer Res. 2010;70:440‐446. 20068163 10.1158/0008-5472.CAN-09-1947

[22] Chen L , Pan J . Dual cyclin‐dependent kinase 4/6 inhibition by PD‐0332991 induces apoptosis and senescence in oesophageal squamous cell carcinoma cells. Br J Pharmacol. 2017;174:2427‐2443. 28444744 10.1111/bph.13836 PMC5513862

[23] Chen W , Li Y , Bao T , Gowd V . Mulberry fruit extract affords protection against ethyl carbamate‐induced cytotoxicity and oxidative stress. Oxid Med Cell Longev. 2017;2017:1594963. 28819542 10.1155/2017/1594963 PMC5551560

[24] Wang Y , Liu M , Jin Y , et al. In vitro and in vivo anti‐uveal melanoma activity of JSL‐1, a novel HDAC inhibitor. Cancer Lett. 2017;400:47‐60. 28455241 10.1016/j.canlet.2017.04.028

[25] Chen L , Li M , Li Q , et al. DKK1 promotes hepatocellular carcinoma cell migration and invasion through beta‐catenin/MMP7 signaling pathway. Mol Cancer. 2013;12:157. 24325363 10.1186/1476-4598-12-157 PMC4029244

[26] Chen RS , Song YM , Zhou ZY , et al. Disruption of xCT inhibits cancer cell metastasis via the caveolin‐1/beta‐catenin pathway. Oncogene. 2009;28:599‐609. 19015640 10.1038/onc.2008.414

[27] Fung TM , Ng KY , Tong M , et al. Neuropilin‐2 promotes tumourigenicity and metastasis in oesophageal squamous cell carcinoma through ERK‐MAPK‐ETV4‐MMP‐E‐cadherin deregulation. J Pathol. 2016;239:309‐319. 27063000 10.1002/path.4728

[28] Zang M , Gong J , Luo L , et al. Characterization of Ser338 phosphorylation for Raf‐1 activation. J Biol Chem. 2008;283:31429‐31437. 18775988 10.1074/jbc.M802855200 PMC2581588

[29] Liu F , Cheng Z , Li X , et al. A novel Pak1/ATF2/miR‐132 signaling axis is involved in the hematogenous metastasis of gastric cancer cells. Mol Ther Nucleic Acids. 2017;8:370‐382. 28918037 10.1016/j.omtn.2017.07.005 PMC5537170

[30] de Castro Junior G , Segalla JG , de Azevedo SJ , et al. A randomised phase II study of chemoradiotherapy with or without nimotuzumab in locally advanced oesophageal cancer: NICE trial. Eur J Cancer. 2018;88:21‐30. 29179134 10.1016/j.ejca.2017.10.005

[31] Zhou J , Wu Z , Wong G , et al. CDK4/6 or MAPK blockade enhances efficacy of EGFR inhibition in oesophageal squamous cell carcinoma. Nat Commun. 2017;8:13897. 28059068 10.1038/ncomms13897 PMC5227099

[32] Li Y , Li P‐K , Roberts MJ , Arend RC , Samant RS , Buchsbaum DJ . Multi‐targeted therapy of cancer by niclosamide: A new application for an old drug. Cancer Lett. 2014;349:8‐14. 24732808 10.1016/j.canlet.2014.04.003 PMC4166407

[33] Chong CR , Sullivan DJ Jr . New uses for old drugs. Nature. 2007;448:645‐646. 17687303 10.1038/448645a

[34] Peng K , Kou L , Yu L , et al. Histone demethylase JMJD2D interacts with beta‐catenin to induce transcription and activate colorectal cancer cell proliferation and tumor growth in mice. Gastroenterology. 2019;156:1112‐1126. 30472235 10.1053/j.gastro.2018.11.036

[35] Kwon Y‐J , Petrie K , Leibovitch BA , et al. Selective inhibition of SIN3 corepressor with avermectins as a novel therapeutic strategy in triple‐negative breast cancer. Mol Cancer Ther. 2015;14:1824‐1836. 26078298 10.1158/1535-7163.MCT-14-0980-T PMC4529816

[36] Radu M , Semenova G , Kosoff R , Chernoff J . PAK signalling during the development and progression of cancer. Nat Rev Cancer. 2014;14:13‐25. 24505617 10.1038/nrc3645 PMC4115244

[37] Yang Z , Wang H , Xia L , et al. Overexpression of PAK1 correlates with aberrant expression of EMT markers and poor prognosis in non‐small cell lung cancer. J Cancer. 2017;8:1484‐1491. 28638464 10.7150/jca.18553 PMC5479255

[38] Ching YP , Leong VY , Lee MF , et al. P21‐activated protein kinase is overexpressed in hepatocellular carcinoma and enhances cancer metastasis involving c‐Jun NH2‐terminal kinase activation and paxillin phosphorylation. Cancer Res. 2007;67:3601‐3608. 17440071 10.1158/0008-5472.CAN-06-3994

[39] Shrestha Y , Schafer EJ , Boehm JS , et al. PAK1 is a breast cancer oncogene that coordinately activates MAPK and MET signaling. Oncogene. 2012;31:3397‐3408. 22105362 10.1038/onc.2011.515 PMC3291810

[40] Han J , Wang F , Yuan S‐Q , et al. Reduced expression of p21‐activated protein kinase 1 correlates with poor histological differentiation in pancreatic cancer. BMC Cancer. 2014;14:650. 25182632 10.1186/1471-2407-14-650 PMC4242600

[41] Chen Y , Ye J , Zhu Z , et al. Comparing paclitaxel plus fluorouracil versus cisplatin plus fluorouracil in chemoradiotherapy for locally advanced esophageal squamous cell cancer: a randomized, multicenter, phase III clinical trial. J Clin Oncol. 2019;37(20):1695‐1703. 30920880 10.1200/JCO.18.02122 PMC6638596

[42] Cooper JS , Guo MD , Herskovic A , et al. Chemoradiotherapy of locally advanced esophageal cancer: long‐term follow‐up of a prospective randomized trial (RTOG 85–01). Radiation Therapy Oncology Group. JAMA. 1999;281:1623‐1627. 10235156 10.1001/jama.281.17.1623

